# Novel intranasal vaccine targeting SARS-CoV-2 receptor binding domain to mucosal microfold cells and adjuvanted with TLR3 agonist Riboxxim™ elicits strong antibody and T-cell responses in mice

**DOI:** 10.1038/s41598-023-31198-3

**Published:** 2023-03-21

**Authors:** Dennis Horvath, Nigel Temperton, Martin Mayora-Neto, Kelly Da Costa, Diego Cantoni, Reinhold Horlacher, Armin Günther, Alexander Brosig, Jenny Morath, Barbara Jakobs, Marcus Groettrup, Heinz Hoschuetzky, Jacques Rohayem, Jan ter Meulen

**Affiliations:** 1 Division of Immunology, Department of Biology, University of Konstanz, Konstanz, Germany; 2 Centre for the Advanced Study of Collective Behaviour, University of Konstanz, Konstanz, Germany; 3 Viral Pseudotype Unit, Medway School of Pharmacy, University of Kent, Canterbury, UK; 4 trenzyme GmbH, Konstanz, Germany; 5 nanoTools Antikoerpertechnik GmbH & Co. KG, Teningen, Germany; 6 Riboxx Pharmaceuticals, Radebeul, Dresden, Germany and Institute of Virology, Dresden University of Technology, Dresden, Germany; 7 Institute of Virology, Philipps University Marburg, Marburg, Germany

**Keywords:** Biotechnology, Immunology, Microbiology

## Abstract

SARS-CoV-2 continues to circulate in the human population necessitating regular booster immunization for its long-term control. Ideally, vaccines should ideally not only protect against symptomatic disease, but also prevent transmission via asymptomatic shedding and cover existing and future variants of the virus. This may ultimately only be possible through induction of potent and long-lasting immune responses in the nasopharyngeal tract, the initial entry site of SARS-CoV-2. To this end, we have designed a vaccine based on recombinantly expressed receptor binding domain (RBD) of SARS-CoV-2, fused to the C-terminus of *C. perfringens* enterotoxin, which is known to target Claudin-4, a matrix molecule highly expressed on mucosal microfold (M) cells of the nasal and bronchial-associated lymphoid tissues. To further enhance immune responses, the vaccine was adjuvanted with a novel toll-like receptor 3/RIG-I agonist (Riboxxim™), consisting of synthetic short double stranded RNA. Intranasal prime-boost immunization of mice induced robust mucosal and systemic anti-SARS-CoV-2 neutralizing antibody responses against SARS-CoV-2 strains Wuhan-Hu-1, and several variants (B.1.351/beta, B.1.1.7/alpha, B.1.617.2/delta), as well as systemic T-cell responses. A combination vaccine with M-cell targeted recombinant HA1 from an H1N1 G4 influenza strain also induced mucosal and systemic antibodies against influenza. Taken together, the data show that development of an intranasal SARS-CoV-2 vaccine based on recombinant RBD adjuvanted with a TLR3 agonist is feasible, also as a combination vaccine against influenza.

## Introduction

Worldwide, SARS-CoV-2 infections have now surpassed 590 million and COVID-related deaths 6.44 million (WHO, August 2022). Several mRNA or adenoviral vector-based vaccines have received full or emergency authorization and are being rolled out in many countries. These vaccines have shown very high efficacy in protecting against hospitalization and death from Covid-19, and 67 to > 94% efficacy against infection in phase 3 clinical trials^1^. However, since all but one recently approved inhalable vaccine are administered via intramuscular injection, they do not induce significant levels of mucosal antibodies in the nasopharyngeal tract, the site of initial viral replication which limits the impact of these vaccines on the spread of the virus in the community. Breakthrough infections with especially the delta variants of SARS-COV-2 (B.1.617.2 and AY.1 sub-lineages) were associated with high levels of viral replication in the respiratory tract and high transmission rates to both to vaccinated and unvaccinated persons^2,3^. Natural antibody-mediated protection for SARS-CoV-2 is likely to last for only 1–2 years and therefore, if vaccine-induced antibodies follow a similar course, regular booster doses will likely be required as long as the virus circulates in the human population^4^. In observational studies of natural SARS-CoV-2 infection, higher levels of nasal antibodies directed against the RBD or spike protein were associated with a lower viral load, and the resolution of systemic symptoms^5^. To this end, efforts to develop nasal SARS-CoV-2 vaccines using different modalities, such as adenoviral vectors expressing recombinant spike protein, are currently underway and potentially attractive vaccine platforms to control the spread of COVID-19 in the community^6^. Importantly, any viable intranasal SARS-CoV-2 vaccine will be expected to generate protection against systemic disease at the same levels as the currently approved vaccines.

The RBD of the SARS-CoV-2 spike is an attractive vaccine antigen because most neutralizing antibodies cloned from human patients are directed against it, especially against epitopes involved in binding to the ACE2 (acetylcholine esterase 2) receptor^7^. Induction of mucosal immune responses requires transport of antigens across the epithelial barrier via specialized phagocytic cells called microfold cells (M-cells), and uptake and processing of antigens by resident macrophages and dendritic cells in the context of activating signals, such as pathogen associated molecular patterns^8^. In the airways, M-cells are found in the epithelium overlying the nasopharynx associated lymphoid tissues (NALT) and express claudins on their basolateral surface, which are ~ 23-kDa four-α -helical transmembrane proteins that assemble into “tight-junction strands” to seal the intercellular space. Targeting M-cells with polypeptides binding to claudin-4, such as the C-terminus of *Clostridium perfringens* enterotoxin (cCPE) fused to vaccine antigens has been shown to increase local and systemic immune responses against influenza, pneumococcus and other pathogens^9–11^. We therefore hypothesize that a targeting SARS-CoV-2 RBD to M-cells in the NALT will increase the immunogenicity of the vaccine.

Previous efforts to develop intranasal vaccines based on soluble recombinant proteins have demonstrated that without adjuvants neither sustained local nor systemic immune responses can be generated^12,13^. Toll-like receptor 3 (TLR3), which is activated by the double stranded RNA intermediates of respiratory RNA viruses, is a potentially attractive target for adjuvants because nasal epithelial cells express TLR3^14^. TLR3 agonists have been used previously as safe adjuvants for intranasal immunization of human volunteers with trivalent influenza vaccine or as monotherapy post-exposure prophylaxis given up to 48 h post challenge with influenza or rhinovirus^15,16^. We used the novel synthetic double stranded RNA (dsRNA) adjuvant Riboxxim™, a TLR3/RIG-I ligand, characterized by its well-defined chemical structure with a nucleotide composition of 100 bp for effective TLR3 triggering and strong activation of human dendritic cells^17,18^. Riboxxim exhibits very good solubility in water and prolonged stability in solution at 4 °C and in serum.

The novel RBD-based intranasal vaccine presented here induced strong mucosal and systemic neutralizing antibody responses against SARS-CoV-2 Wuhan strain, as well as alpha, beta and delta variants, both when used for intranasal prime/intranasal boost as well as for subcutaneous prime/intranasal boost in mice. Robust CD4 and CD8 t-cell responses were induced as well. Lastly, the platform lends itself to adding additional antigens, such as influenza hemagglutinin (HA), which raises the prospect of developing combination vaccines against Covid and other respiratory infections.

## Results

### Recombinant SARS-CoV-2 receptor binding domain retains conformational epitopes and is stable at 4 °C and − 80 °C

Conformational stability of recombinant RBD or RBD-cCPE were assessed by ELISA using recombinant ACE2 protein, and the conformationally sensitive recombinant human anti-RBD monoclonal antibody CR3022^19^. Purified recombinant RBD and RBD-cCPE were recognized by ACE2 and CR3022, with and without Riboxxim, and stable at 4 °C and at − 80 °C for at least 2 months without loss of activity (Suppl. Figure 1).

### SARS-CoV-2 RBD-cCPE vaccines induce RBD and spike binding IgA and IgG antibodies

**Figure 1:**
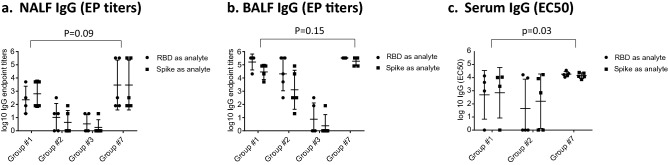
Monovalent SARS-CoV-2 RBD vaccines induce IgG antibody responses in NALF, BALF and serum. Mice were prime-boost immunized (d0, d21) intranasally with non-targeted (nt) RBD, strain Wuhan-Hu-1 (group #1) or primed subcutaneously and boosted intranasally with nt RBD (group #2), or prime-boosted intranasally with nt RBD, adjuvanted with Riboxxim and Chitosan (group #3), or prime-boosted intranasally with M-cell targeted RBD (RBD-cCPE, group #7). All vaccines were adjuvanted with Riboxxim. Sera, nasal fluid (NALF) and bronchioalveolar fluid (BALF) were obtained on day 28, 1 week after the boost, diluted in fourfold steps, and assayed for IgA antibodies in a Luminex assay with recombinant nt RBD, or recombinant trimeric spike protein, both derived from SARS-CoV-2 strain Wuhan-Hu-1. Endpoint titers (EP) were defined as highest sample dilution exceeding the signal of mean of control group (#9) + 3xSD. Log10 EC_50_ values were calculated using GraphPad Prism 7, 5PL. For mice with low titers, EC_50_ could not be determined and log10 EC_50_ (IgG) was accepted as zero. Log10 of endpoint titers or EC_50_ were plotted with geometric mean ± geometric SD. Two-way comparisons using the Mann–Whitney U test.

Mice were primed on d0, boosted on d21 with different vaccine formulations and NALF, BALF and serum were obtained 7 days post-boost immunization (d28) (Table 1). IgA and IgG antibodies binding to recombinant monomeric SARS-CoV-2 RBD or trimeric SARS-CoV-2 spike protein were determined using Luminex or ELISA, respectively, with no significant differences observed in antibody titers against the antigens. No specific antibodies were detectable in sera of mice from groups #4 (RBD, no Riboxxim), group #5 (Riboxxim only), group #6 (Riboxxim and Chitosan only), group #8 (RBD-cCPE), and group #9 (PBS control) (data not shown). Antibodies were detected in serum, NALF and BALF of all animals immunized with RBD plus Riboxxim (group #1, Fig. 1a–c), or RBD-cCPE plus Riboxxim (group #7, Fig. 1a–c), and also in all animals who were primed subcutaneously and boosted intranasally with RBD plus Riboxxim (group #2, Fig. 1a–c). Highest antibody titers were observed in animals immunized with RBD-cCPE adjuvanted with Riboxxim, followed by adjuvanted RBD. Supplementary Figure 2a shows the superior immunogenicity (seroconversion, GMT) of RBD-cCPE over RBD, which resulted in IgG-GMT that were 11.9-fold, 9.9-fold and 5.7-fold higher in serum, BALF, and NALF, respectively, compared to RBD. In a linear regression model, RBD-cCPE was the most important predictor (*p* = 0.39 for NALF, *p* = 0.032 for BALF, *p* = 0.063 for serum. Supplementary Figure 2a). Variability of IgG titers was high in NALF for all vaccine groups (endpoint titer range 0–327,680 Fig. 1a), in BALF for groups #1, #2 and #3 (Fig. 1b), and in serum for groups #1 and #2 (Fig. 1c). In contrast, variability was low between animals of group #7 in BALF and in serum (Fig. 1b, c), with highest titers observed in the latter. In serum, EC50 titers for group #7 were approx. 4E3 (range 11,059–36,581), with EP titers exceeding 3E6; in BALF EC50 titers for group #7 approached 3E3, with EP titers exceeding 3E5 (Fig. 1b, c).

**Table 1:** Groups of mice immunized with different formulations of monovalent SARS-CoV-2 RBD vaccines.

Group #	Riboxxim™ (μg)	Chitosan (μg)	RBD (μg)	RBD-C-CPE (μg)
1	50	–	10	–
2	50	–	10	–
3	50	10	10	–
4	–	–	10	–
5	50	–	–	–
6	50	10	–	–
7	50	–	–	10
8	–	–	–	10
9 (PBS)	–	–	–	–

Nine groups of mice (n = 5 animals per group) were immunized with monovalent SARS-CoV-2 RBD vaccines of the indicated formulations. RBD-cCPE: receptor binding domain fused to the C-terminal polypeptide of the *C. perfringens* toxin (cCPE), which targets Claudin-4 on microfold (M) cells. Riboxxim™: Synthetic, double-stranded RNA TLR3/RIG-I agonist.

**Figure 2:**
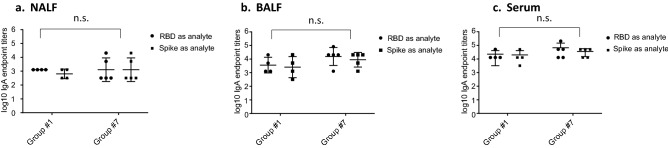
Monovalent SARS-CoV-2 RBD vaccines induce IgA antibody responses in NALF, BALF and serum. Mice were prime-boost immunized intranasally (d0, d21) with non-targeted (nt) RBD, strain Wuhan-Hu-1 (group #1), or M-cell targeted RBD (RBD-cCPE, group #7). Both vaccines were adjuvanted with Riboxxim. Sera were obtained on day 28, 1 week after the boost, diluted in fourfold steps, and assayed for IgG antibodies in Luminex with recombinant nt RBD, or recombinant trimeric spike protein, both derived from SARS-CoV-2 strain Wuhan-Hu-1. Endpoint titers (EP) were defined as highest dilution of a sample exceeding the mean signal of the lowest 2 dilutions + 3SD. Log10 of endpoint titers were plotted with geometric mean ± geometric SD. Two-way comparisons using the Mann–Whitney U test.

IgA responses were only detected in groups #1 and #7, ranging from 3E1 to 2E4 in NALF and BALF (Fig. 2a, b), and from 3E3 to2E5 in serum (Fig. 2c). In NALF, RBD-cCPE/Riboxxim induced 1.7-fold higher GMT titers compared to RBD, in BALF the GMT increase was twofold, and in serum the GMT increase was 3.7-fold (data not shown).

### SARS-CoV-2 RBD-cCPE vaccine induces SARS pseudotype (Wuhan-Hu-1, variants B1.1.7 and B1.351) neutralizing antibodies

**Figure 3:**
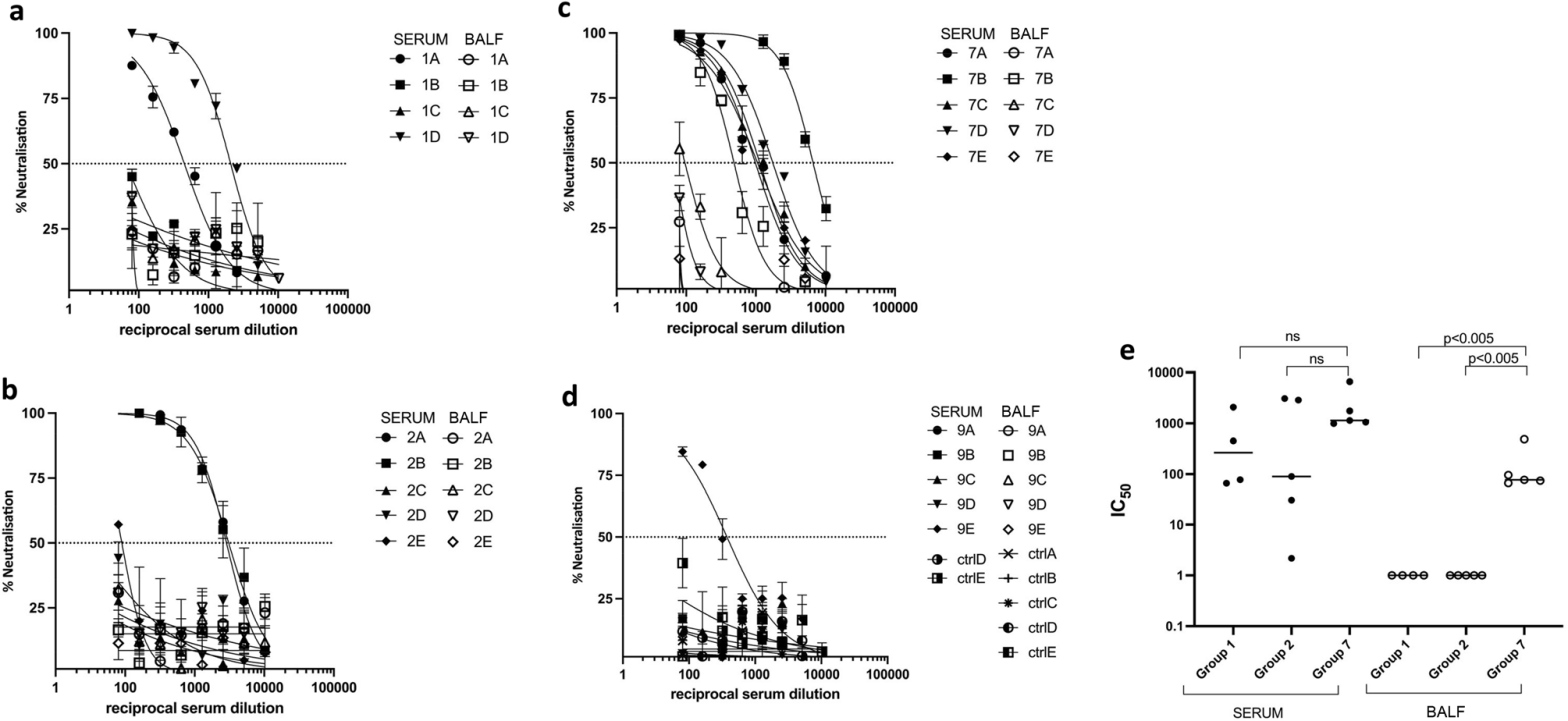
Monovalent SARS-CoV-2 RBD vaccines induce SARS-CoV-2 pseudovirus (Wuhan-Hu-1) neutralizing antibodies in serum and BALF. Mice were prime-boost immunized intranasally (d0, d21) with non-targeted (nt) RBD, strain Wuhan-Hu-1 (group #1), or M-cell targeted RBD (RBD-cCPE, group #7), and sera and BALF obtained on day 28, 1 week after the boost, diluted in tenfold steps, and assayed for neutralization of SARS-CoV-2 pseudoviruses (PV). The spike protein sequence was derived from SARS-CoV-2 strain Wuhan-Hu-1. (**a**) (mice group #1): Serum and BALF, intranasal prime-boost immunization with nt RBD plus Riboxxim. (**b**) (group #2): Serum and BALF, subcutaneous prime-intranasal boost immunization with nt RBD plus Riboxxim. (**c**) (group #7): Serum and BALF, intranasal prime-boost immunization with M-cell targeted RBD (RBD-cCPE) plus Riboxxim. (**d**) (group #9): Serum and BALF, control mice (ctrlA-E). (**e**) In BALF, IC50 of neutralizing antibody was significantly higher for group #7 compared to group #2 or #3, respectively (*p* < 0.005, two-sided Mann–Whitney U test), but not in serum.

**Figure 4:**
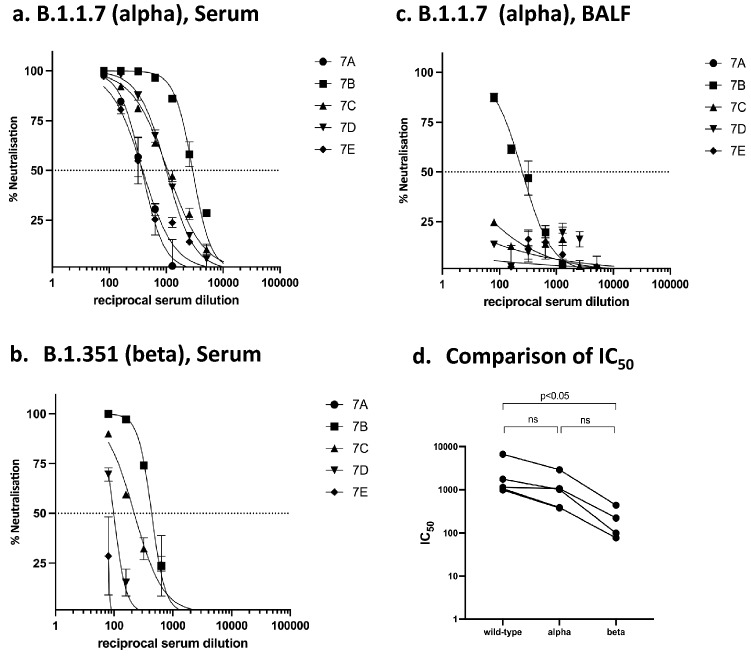
Monovalent SARS-CoV-2 RBD-cCPE vaccine induces neutralizing antibodies against SARS-CoV-2 pseudotypes B.1.1.7 and B.1.351. Mice were prime-boost immunized (d0, d21) intranasally with M-cell targeted RBD (RBD-cCPE, group #7), and sera and BALF obtained on day 28, 1 week after the boost, diluted in tenfold steps, were assayed for neutralization of pseudotype viruses. The spike protein sequence was derived from SARS-CoV-2 strain Wuhan-Hu-1, strain B.1.1.7 (Alpha), or B.1.351 (Beta). (**a**, **b**) Serum neutralization. (**c**) BALF neutralization. (**d**) Comparison of serum neutralization of SARS-CoV-2-PV wildtype (Wuhan-Hu-1), alpha, and beta variants. Statistical analysis (Mann–Whitney U test).

Neutralizing antibody titers were determined in BALF, and sera collected on day 7 post-boost immunization (day 28 post prime) against lentiviral vectors pseudotyped with SARS-CoV-2 spike from Wuhan-Hu-1 (wildtype, WT), and variants B.1.1.7 (Alpha) and B.1.351 (Beta). No neutralization tests were performed on mice from groups #3–6 and #8, because they had no or only low binding antibodies. Neutralizing antibodies were detectable in all sera and two BALF (animals 7b + c) of mice belonging to groups #1 (RBD/Riboxxim), #2 (RBD-cCPE/Riboxxim, subcutaneous prime/intranasal boost), and #7 (RBD-cCPE), with most consistent response (100% seroconversion) and highest IC_50_ titers observed in the mice which received RBD-cCPE together with Riboxxim (Figs. 3a–d, 4a, b). All sera with neutralizing antibodies neutralized WT and B.1.1.7 variant, with similar IC_50_ titers, whereas IC_50_ titers were approx. tenfold lower for the B.1.351 variant (Fig. 4a–d). Figures 3a, b and 4a, b depict results of independent experiments. For BALF, the observed IC_50_ differences between group #7 (RBD-cCPE, intranasal prime/intranasal boost) and #1 (RBD, i.n/i.n.) and #2 (RBD, subcutaneous prime/i.n. boost) reached significance (*p* < 0.05, Fig. 3e). Due to the small number of animals and variability of responses the observed differences in serum IC50 between group #7 and #1, and #7 and #2, respectively, did not reach significance at an alpha level of 0.05.

### Bivalent SARS-CoV-2 RBD-cCPE / H1 influenza HA_1_-cCPE vaccine induces RBD, spike and HA1 binding, and SARS pseudotype neutralizing antibodies

**Figure 5:**
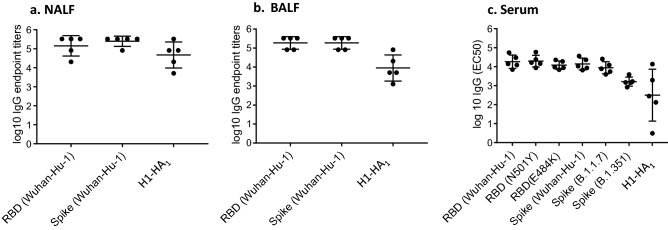
Bivalent SARS-CoV-2 RBD-cCPE/Influenza H1-HA_1_-cCPE vaccine induces IgG antibody responses in NALF, BALF and serum against RBD and spike of SARS-CoV-2 wildtype and variants. Mice were prime-boost immunized intranasally (days 0 and 21) with bivalent SARS-CoV-2 RBD-cCPE/influenza H1-HA_1_-cCPE vaccine, adjuvanted with Riboxxim. NALF, BALF and sera were obtained on day 28, 1 week after the boost, diluted in fourfold steps, and assayed for IgG antibodies in Luminex with recombinant monomeric RBD (Wuhan-Hu-1/wildtype, or point mutations N501Y, or E484K), or recombinant trimeric spike protein (Wuhan-Hu-1, B.1.1.7, B.1.351), or recombinant monomeric H1-HA_1_ (A/swine/Guangxi/3843/2011(H1N1), respectively. Endpoint titers (NALF, BALF) or EC_50_ (serum) were calculated where applicable. Log10 of endpoint titers or EC_50_ were plotted with geometric mean ± geometric SD.

**Figure 6:**
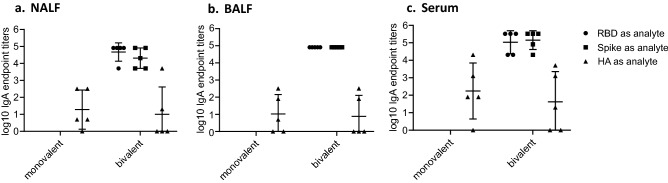
Monovalent influenza H1-HA_1_ and bivalent SARS-CoV-2 RBD/influenza H1-HA_1_ vaccines induce IgA antibody responses in NALF, BALF and serum. Mice (n = 5/group) were prime-boost immunized intranasally (days 0 and 21) with monovalent influenza H1-HA_1_-cCPE or bivalent SARS-CoV-2 RBD-cCPE/influenza H1-HA_1_-cCPE vaccine, adjuvanted with Riboxxim. NALF, BALF and sera were obtained on day 28, 1 week after the boost, diluted in fourfold steps, and assayed for IgA antibodies by ELISA with recombinant monomeric RBD (Wuhan-Hu-1/wildtype), recombinant trimeric spike protein, or recombinant monomeric H1-HA_1_ (A/swine/Guangxi/3843/2011(H1N1), respectively. Log10 of endpoint titers were plotted.

NALF, BALF and serum of mice obtained on day 28 post prime immunization (day 7 post boost) with bivalent SARS-CoV-2/Influenza vaccine were assayed for IgG and IgA antibodies for binding to recombinant RBD (Wuhan-Hu-1/wildtype, or RBD point mutations N501Y, or E484K), or recombinant trimeric spike protein (WT, variants B.1.1.7/alpha and B.1.351/beta), or recombinant H1-HA_1_ (consensus sequence based on H1N1/swine/Guangxi/3843/2011), using Luminex technology for IgG and ELISA for IgA, respectively. High IgG antibody titers with similar binding to SARS-CoV-2 RBDs (WT, point mutants), WT and mutated spike-trimers were detectable in NALF, BALF and serum, with EP titers in the 1E4 to 5E5 range (Fig. 5a–c). IgG antibodies against recombinant monomeric H1-HA_1_ were detected in all three body fluids, with highest titers in NALF and BALF (Fig. 5a–c). Similarly, IgA antibody reactive with RBD and spike was detected in NALF, BALF and serum, with EP ranging from 1E4 to 1E6 (Fig. 6a–c). IgA antibodies specific for H1-HA_1_ were detectable in NALF, BALF and serum, with EP titers ranging from 1E1 to 1E4 (Fig. 6a–c).

**Figure 7:**
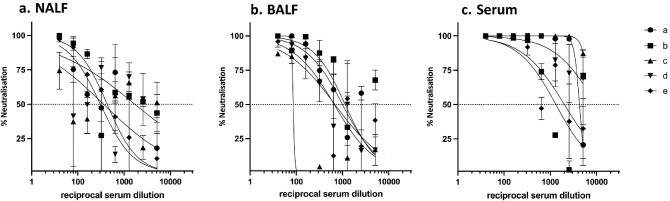
Bivalent SARS-CoV-2 RBD-cCPE/influenza H1-HA_1_-cCPE induces SARS-CoV-2 pseudovirus (Wuhan-Hu-1) neutralizing antibodies in NALF, BALF and serum. Mice (n = 5, a–e) were prime-boost immunized intranasally (days 0 and 21) with bivalent SARS-CoV-2 RBD-cCPE/influenza H1-HA_1_-cCPE vaccine, adjuvanted with Riboxxim. NALF, BALF and sera were obtained on day 28, 1 week after the boost, diluted in fourfold steps, and assayed for neutralization of SARS-CoV-2 pseudoviruses. The spike protein sequence was derived from SARS-CoV-2 strain Wuhan-Hu-1.

**Figure 8:**
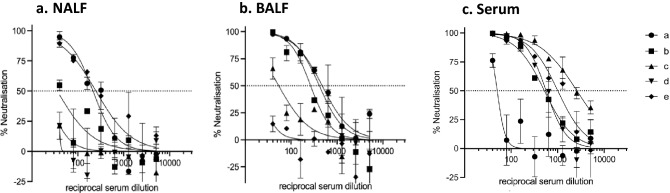
Bivalent SARS-CoV-2 RBD-cCPE/influenza H1-HA_1_-cCPE induces SARS-CoV-2 pseudovirus (Delta variant B.1.617.2) neutralizing antibodies in NALF, BALF and serum. Mice (n = 5) were prime-boost immunized intranasally (days 0 and 21) with bivalent SARS-CoV-2 RBD-cCPE/Influenza H1-HA_1_-cCPE vaccine, adjuvanted with Riboxxim. NALF, BALF and sera were obtained on day 28, 1 week after the boost, diluted in tenfold steps, and assayed for neutralization of SARS-CoV-2 pseudoviruses. The spike protein sequence was derived from SARS-CoV-2 strain B.1.617.2.

Neutralizing antibodies against WT Wuhan-Hu-1 SARS-CoV-2 PV were detectable in NALF (range 0–175), BALF (86–1141) and serum (1320 to > 5120) serum of all animals (Fig. 7a–c) and against the variant B.1.617.2 (Delta) in 2/5 NALF, 3/5 BALF and in 4/5 sera with similar IC_50_ titers as against the wildtype (Fig. 8a–c).

### Monovalent SARS-CoV-2 RBD-cCPE and bivalent SARS-CoV-2 RBD-cCPE/influenza H1-HA_1_-cCPE vaccines induce strong CD4 and CD8 T-cell responses

**Figure 9:**
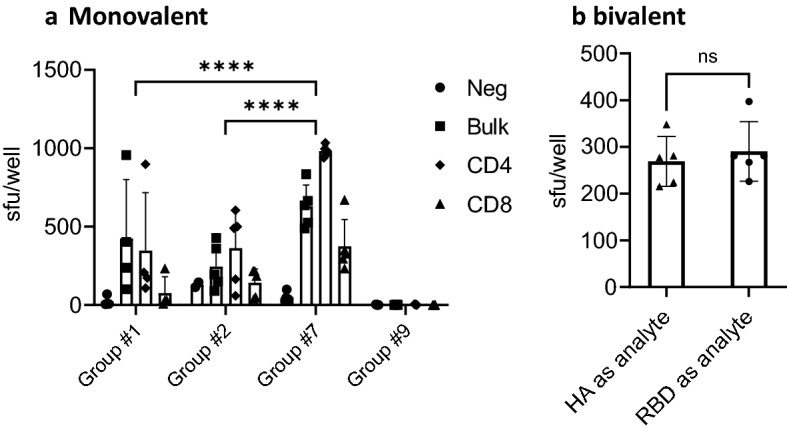
Monovalent SARS-CoV-2 RBD-cCPE and bivalent SARS-CoV-2 RBD-cCPE/Influenza H1-HA_1_-cCPE vaccines induces CD4 and CD8 T-cell responses against recombinant SARSCoV-2 RBD or influenza H1-HA_1_. Monovalent SARS-CoV-2 RBD-cCPE and bivalent SARS-CoV-2 RBD-cCPE/Influenza H1-HA_1_-cCPE vaccines induce CD4 and CD8 T-cell responses against recombinant SARS-CoV-2 RBD or influenza H1-HA_1_ protein. (**a**) Mice were prime-boost immunized intranasally (days 0, 21) with either monovalent non-targeted RBD, strain Wuhan-Hu-1 (group #1), M-cell targeted RBD (RBD-cCPE, group #7), both adjuvanted with Riboxxim or PBS (group #9). On day 28 bulk splenocytes (Bulk, squares) or splenocytes MACS-depleted for either CD8 (CD4, rhombus) or MACS-depleted for CD4 (CD8, triangles) were analyzed for IFNγ production upon restimulation with recombinant RBD or recombinant ACE2 as control (Neg control, dots) in ELISPOT. Data are presented as means + SD. Statistics: two-way ANOVA followed by Tukey’s multiple comparisons test with *ns* not significant; ***p ≤ 0.001; ****p < 0.0001. The individual CD4 and CD8 T-cell responses were statistically significantly different between group #1 and #7 (*p* = 0.0018 and *p* = 0.0051, respectively) and between group #2 and group #7 (*p* = 0.0014 and *p* = 0.0203, respectively), by one-way ANOVA. (**b**) Mice were prime-boost immunized intranasally with bivalent SARS-CoV-2 RBD-cCPE/influenza H1-HA_1_-cCPE, adjuvanted with Riboxxim. On day 28 splenocytes were analyzed for IFNγ production upon restimulation with recombinant RBD (dots) derived from SARS-CoV-2 strain Wuhan-Hu-1 or an H1-HA_1_ consensus sequence (squares) protein in ELISPOT. Data are presented as means + SD. Statistics: Unpaired *t* test, *ns* not significant.

In addition to the generation of neutralizing antibodies, the systemic T cell response against the SARS-CoV-2 RBD and influenza H1-HA_1_ was analyzed for monovalent and bivalent vaccine formulations. On day 28 post prime (7 days post boost), splenocytes were isolated and antigen specific T cell responses measured by IFNγ secretion in ELISPOT. For monovalent vaccines, strong IFNγ secretion in response to stimulation with RBD were detectable for both CD4^+^ and CD8^+^ T cells in all vaccinated groups, with highest number of spot forming units observed in mice from group #7, ranging for CD4^+^ and CD8^+^ T-cells from 939 to 1034/1E6 and 233 to 670/1E6 splenocytes, respectively. While T-cell responses between groups #1 and #2 were not statistically significantly different, T-cell responses in group #7 (RBD-cCPE) were significantly higher than those in groups #1 (RBD) and #2 (RBD, subcutaneous prime/intranasal boost, Fig. 9a, b). CD4 and CD8 T-cell responses were statistically significantly different between group #7 and #1 (*p* = 0.0018 and *p* = 0.0051, respectively) and between group #7 and group #2 (*p* = 0.0014 and *p* = 0.0203, respectively), but not between groups # 1 and #2, by one-way ANOVA. A bivalent SARS-CoV-2 RBD-cCPE/influenza H1-HA_1_-cCPE vaccine adjuvanted with Riboxxim induced similarly potent T cell responses against both the RBD and H1-HA_1_,

## Materials and methods

### Production of recombinant SARS-CoV-2 receptor binding domains, influenza H1-HA_1_ antigen, and human ACE2

The SARS-CoV-2 Spike S1 (RBD) target sequence (AA 319-541) was synthesized as gBLOCK gene fragment (Integrated DNA Technologies, Inc) containing a C-terminal octa-histidine tag and cloned into expression vector pTZ_M_24 (trenzyme GmbH) containing an optimized secretion signal. The SARS-CoV-2 Spike S1 (RBD)—fusion protein containing AA 319-541 of SARS-CoV-2 Spike S1 (RBD) fused at its C-terminus via a short linker sequence (GGGGS) to AA 194-319 of *Clostridium perfringens* heat-labile enterotoxin B chain (cCPE) was synthesized as described above and cloned into expression vector pTZ_M_26 (trenzyme GmbH) containing an optimized secretion signal followed by a nona-histidine tag. The hACE2 target sequence (AA 20-708) was synthesized as described and cloned into expression vector pTZ_M_27a containing an optimized N-terminal secretion signal and a C-terminal TEV-cleavable octa-histidine tag. Recombinant H1-HA_1_ was synthesized based on a codon-optimized consensus sequence of isolates of the avian-like A/swine/Guangxi/3843/2011 (H1N1) influenza virus^20^ (Gene Bank Acc #KM028359) and cloned into expression vector pTZM26, providing an optimized secretion signal followed by a TEV-cleavable nona-histidine tag.

The plasmids were transiently transfected into serum free HEK293 cells (trenzyme GmbH) by use of FectoPro (Polyplus-transfection SA). Cells were cultivated in Erlenmeyer flasks of appropriate size at 37 °C, 8% CO2 shaking at 150 rpm for 4 days. Supernatants were clarified by centrifugation prior to loading onto the columns. The RBD proteins were IMAC purified by Ni Sepharose® excel resin (Cytiva). After washing with Phosphate buffer (pH 7.5) containing 500 mM NaCl and 10 mM imidazol, the bound (target) protein(s) were/was eluted using the same buffer containing 500 mM imidazol and relevant fractions were pooled after analysis by SDS-PAGE. The hACE2 protein was purified by ion exchange chromatography using Q-Sepharose FF (Cytiva) resin: after dilution of cell culture supernatant with equilibration buffer (50 mM Tris; pH 8.0) bound proteins were eluted by a linear gradient 0–100% buffer B (50 mM Tris, 400 mM NaCl; pH 8.0) over a volume of 30 CV (column volumes). hACE2 protein containing fractions were identified by SDS-PAGE and pooled accordingly. Following this purification step, the buffer of all proteins was exchanged to PBS, pH 7.4 using a HiPrep 26/10 desalting column (Cytiva) and proteins were aliquoted, (flash-) frozen and stored at − 80 °C.

### Functional ELISA

ELISA plates (Nunc maxisorp) were coated with 200 ng recombinant hACE2/well in coating buffer (50 mM NaHCO3 pH9) over night at 4 °C. A standard sandwich ELISA protocol was performed using PBS with 3% BSA as blocking agent or with 0.05%Tween-20 as wash buffer. Serial dilutions of either purified RBD, or RBD or RBD-cCPE formulations containing Riboxxim™ (Riboxx Pharmaceuticals, Dresden), representative of the vaccine formulations used in the animal studies, were loaded to appropriate wells and incubated for 2 h at RT. Monoclonal antibody CR3022 (Cat# Ab01680-23.0, Absolute Antibody Ltd) was used at concentration of 0.1 µg/ml for detection of RBD, followed by addition of Goat-anti-Rabbit HRP (Cat#S2438, Sigma-Aldrich Chemie GmbH), staining with 100 µl TMB (Cat#34028, Thermo Fisher Scientific). The reaction was stopped with stop with 50 µl H_2_SO_4_ (50 µl, 2 M). Standard sandwich ELISA was performed using the same protocol but with coat antibody (Cat# 40150-D003, Sino Biological Europe GmbH, 25 ng/well) and detection antibody (HRP conugate antibody Cat# 40150-D001-H, Sino Biological Europe GmbH), followed by staining with TMB and stop with H_2_SO_4_. OD_450nm_ was determined using a Wallac Victor V multilabel reader (Perkin Elmer).

### Riboxxim synthesis

The TLR3/RIG-I of Riboxxim™ was manufactured using a proprietary process as described previously^17^. Briefly, this process is an isothermal polymerization using an RNA-dependent RNA-polymerase and a single stranded RNA template, which yields perfectly annealed double stranded RNA consisting of 100 bp. GMP-grade Riboxxim was stored at 4 °C in water for injection at a concentration of 1 mg/ml.

### Preparation of different formulations of monovalent SARS-CoV-2 RBD vaccines, monovalent influenza H1-HA_1_ and bivalent SARS/influenza H1-HA_1_ vaccines

Recombinant RBDs and Riboxxim were co-formulated in water for injection either as monovalent SARS vaccines, or as monovalent influenza or bivalent SARS/influenza vaccines. Table 1 shows the different vaccine formulations and immunization routes for monovalent SARS-CoV-2 vaccines (mice groups #1–9). For monovalent influenza immunization n = 5 mice were immunized with 10 µg influenza H1-HA1-cCPE antigen plus 10 µg Riboxxim, and for bivalent vaccination with each 10 µg of SARS-CoV-2-RBD-cCPE and H1-HA_1_-cCPE, plus 10 µg of Riboxxim.

For chitosan-adjuvantation, RBD and Riboxxim were co-formulated together with a 0.4 mg/ml chitosan solution. Since intranasally administered vaccines must be transported over only a very small distance, remain in the nose only for about 15 min and are not exposed to low pH values, they do not necessarily have to be incorporated into chitosan microparticles, but may also be co-administered as chitosan solution^21^.

### Animal immunization

For immunization studies 8–10 weeks old female BALB/c mice were immunized either subcutaneously at the base of the tail or intranasally under isoflurane anesthesia. Intranasal prime (day 0) and boost (day 21) vaccinations were performed by instilling 25 µl of vaccine into each nostril. For systemic prime/intranasal boost, 50 µl of vaccine were injected subcutaneously on day 0, and 25 µl were instilled into each nostril on day 21. Nasal instillation of the vaccine was done under short (3–5 min) isoflurane anesthesia. One week after the boost immunization mice were sacrificed, the trachea was cannulated, and serum and target organs were collected for further analysis. The BALF was obtained by flushing the lungs with 800 µl PBS. The NALF was obtained by flushing the nostrils through the trachea with 400 µl PBS using an 18 G needle.

### Determination of SARS-CoV-2 spike and RBD binding murine IgG antibodies by Luminex assay in serum, nasal lavage fluid (NALF) and broncho-alveolar lavage fluid (BALF)

lgG antibody binding to immobilized analyte proteins was monitored by a Luminex bead-based assay, following the manufacturer’s instructions with some proprietary modifications by Nanotools. Antigens used for antibody detection were recombinant RBD or trimeric spike protein derived from SARS-CoV-2, strain Wuhan-Hu-1 (trenzyme, Konstanz)*.* Briefly, analyte proteins were conjugated to carboxylated Luminex beads via carbodiimide chemistry (nanoTools, proprietary protocol). In order to enable multiplexing in Luminex assays, each analyte protein was assigned a unique bead number, starting with 1000–2000 beads per assay point. All assays and washing steps were done in buffer V30 (nanoTools proprietary buffer). Mouse sera were initially diluted 1:200 and BALF and NALF 1:10, respectively, and then further diluted 8 times in fourfold steps. 100 µl of each serum/BALF/NALF dilution was mixed with 100 µl of a suspension containing beads conjugated to monomeric RBD or trimeric spike protein, respectively, then incubated for 1 h at room temperature, centrifuged and washed. In order to determine target specific IgG titers, the beads were then incubated with 1 µg/ml biotinylated subclass-specific goat anti mouse Ig in V30 buffer for 1 h at room temperature and washed. Beads were resuspended in 1 µg/ml fluorochrome labelled Streptavidin in V30 buffer, incubated 1 h at room temperature, washed and resuspended in 140 µl Luminex read buffer. Samples were analysed with Bioplex 100/200 instrumentation.

Endpoint titers (EP) were defined as highest sample dilution exceeding the signal of the mean of the control group (#9) + 3 × standard deviation (SD). Log10 EC50 values were calculated using GraphPad Prism 7, using the 5-parameter logistic function (5PL). For mice with low titers, EC50 could not be determined and log10 EC50 (IgG) was accepted as zero. Log10 of endpoint titers or EC50 were plotted with geometric mean ± geometric SD.

### Determination of SARS-CoV-2 spike and RBD binding murine IgA antibodies by ELISA assay in serum, NALF and BALF.

RBD specific murine IgA antibody titers were determined by ELISA. Briefly, ELISA plates (Greiner) were coated with 20 µg/ml of recombinant RBD or trimeric spike protein derived from SARS-CoV-2, strain Wuhan-Hu-1 overnight at 4 °C in PBS. Serial dilutions of mouse sera, BALF and NALF were prepared and loaded on the precoated plates at RT for 2 h. For detection, ALP conjugated anti-mouse IgA mAb (MT39A-ALP, MabTech) was used according to the manufacturer’s instructions followed by staining with 100 µl pNPP ELISA substrate (MabTech) for 1 h at RT. OD_405nm_ was determined using an infinite M200PRO plate reader (TECAN). Endpoint titers were defined as highest sample dilution exceeding the signal of the mean of the control group + 3 × standard deviation (SD).

### Determination of RBD specific T-cell responses by IFNɣ ELISPOT assay in splenocytes

Frozen splenocytes of immunized mice were rapidly thawed, and the freezing medium was diluted into 14 ml of IMDM media (gibco), centrifuged and resuspended in 10 ml of fresh IMDM media, supplemented with 10% FBS (gibco), 100 U/ml Penicillin and 100 µg/ml Streptomycin. Freshly thawed or freshly isolated splenocytes were incubated at 37 °C for 1 h, followed by centrifugation and resuspension in fresh media before being counted. Splenocytes were depleted of CD4^+^ or CD8^+^ T cells using MACS CD4 or CD8a microbeads (Miltenyi Biotech) according to the manufacturer’s instructions. IFNγ production of SARS-CoV-2-Spike-RBD specific cells or HA_1_ specific cells was analyzed using a commercially available mouse ELISPOT antibody pair (BD Bioscience) according to the manufacturer’s instructions. Briefly, 1 × 10^6^ to 2.5 × 10^5^ splenocytes were incubated in pre coated MultiScreen®HTS PVDF Filter Plates (Merck Millipore) in triplicates and re-stimulated with 100 µg/ml full length recombinant His-tagged SARS-CoV-2-Spike S1 (RBD) or His-tagged H1-HA_1_. Negative controls were incubated with 100 µg/ml recombinant His-tagged hACE2. At 37 °C in a humidified CO2 atmosphere for 24 h. Following the incubation with a biotinylated secondary antibody specific for IFNγ, a streptavidin–alkaline phosphatase enzyme conjugate was added. With the addition of the BCIP®/NBT substrate solution (Sigma Aldrich), a purple precipitate is formed as spots at the sites of captured IFNγ. Automated spot analysis and quantification of developed plates were performed using the ImmunoSpot® S6ULTRA analyzer and ImmunoSpot® software version 7.0.33.1 (C.T.L. Europe).

### Pseudotype virus preparation

Production of lentiviral pseudotyped viruses (PV) expressing the spike protein of either wildtype Wuhan-Hu1, or the variants B.1.1.7 (alpha) or B.1.351 (Beta) were carried out as previously described^22^. Briefly, T-75 flasks containing HEK293T/17 cells at 50% confluency were co-transfected with 1 µg of lentiviral Gag-pol p8.91 plasmid, 1.5 µg of lentiviral vector expressing firefly luciferase pCSFLW, and 1 µg of either WT, B.1.1.7 or B.1.351 spike plasmid in pcDNA3.1 + expression vector, using Fugene HD at a mixture ratio of 3:1 (DNA:Fugene HD) in 200 µL Opti-MEM. Transfection mixtures were incubated for 15 min prior to addition into the flasks containing the cells. PVs were then harvested 48 h post transfection by filtering the culture medium through a 0.45-µm cellulose acetate filter. PVs were aliquoted and stored at − 80 °C. PV titers were obtained via a twofold serial dilution, then adding HEK293T/17 cells expressing ACE2/TMPRSS2 at a density of 10,000 cells per well. After 48 h, cells were lysed using BrightGlo (Promega) and luciferase expression was measured using a GlowMax Navigator (Promega) luminometer.

### Serum/plasma pseudotyped virus neutralization assay

PV neutralization assays were carried out as previously described^23^. Briefly, heat inactivated BALF or sera obtained from mice were mixed at a 1:40 dilution in DMEM and a twofold serial dilution was carried out in a 96-well plate to a final dilution of 1:5120. PVs were then added (10^5^ RLU/well) to the wells and plates were incubated at 37 °C for 1 h prior to addition of pre-transfected HEK293T/17 cells expressing ACE2/TMPRSS2 at a cell density of 10,000 cells per well. Plates were incubated for 48 h at 37 °C and 5% CO2. After 48 h, culture media was removed, and luminescence was measured using the Bright-Glo Luciferase assay system (Promega). Neutralization was calculated relative to virus only controls. Dilution curves were presented as a mean neutralization with standard error of the mean (SEM). IC_50_ values were calculated in GraphPad Prism^24^. The IC_50_ within groups were summarized as a geometric mean titer and statistical comparison between groups were made with the two-sided Mann-Whitney U test.

### Animals and ethics statement

BALB/cAnNCrl (BALB/c) mice were originally purchased from Charles River (Sulzfeld, Germany) and were further bred in the accredited animal facility of the University of Konstanz under specific pathogen-free conditions. Mice were kept in air-conditioned rooms with controlled temperature (22 °C), 55% relative humidity, and constant ventilation (17 air changes/h). Animals were provided ad libitum access to standard, autoclaved laboratory animal diet, and tap water. Female mice were used at 8–10 weeks of age. The number of animals in each group was determined according to statistical verification and previous studies. Animal experiments were conducted in compliance with ethical standards of German and EU guidelines after approval by the animal experimentation ethics committee of the Review Board of Governmental Presidium Freiburg, Germany (approval numbers G-20/142, G-21/119), equivalent to the ARRIVE guidelines*.*

### Statistical methods

Two-way comparisons using the Mann–Whitney U test was done for ELISA results (endpoint titers and EC_50_) and a one-way ANOVA to compare IC_50_ of neutralization titers in serum and BALF. ELISPOT counts were compared individually (CD4, CD8) or groupwise (all T-cell responses) by one-sided ANOVA, followed by Tukey’s multiple comparison test for group comparisons. To compare the effect of targeting the RBD to M-cells (RBD-cCPE) on ELISA titers in NALF, BALF and serum, compared to non-targeted RBD, a linear regression model was built, using “compartment” (serum, BALF, NALF) and “antigen” (RBD, RBD-cCPE) as factors, and a two-way comparison at a fixed dilution of 1:1280 was performed, using the Mann–Whitney U test.

## Discussion

First-generation SARS-CoV-2 vaccines based on mRNA or recombinant adenoviral vectors provide a high level of protection against severe disease, hospitalization, and death. However, rapidly waning immunity, clinically relevant break-through infections, especially with emerging variants, and shedding of high levels of virus by asymptomatically infected, fully vaccinated persons are cause for concern with respect to the long-term control of COVID-19^25–28^. In SARS-CoV-2 infection, mucosal antibodies against spike (S) and RBD increase 7–9 days after symptom onset, remain elevated for at least 9 months and were found to be correlated with a lower viral load and a faster decline in systemic symptoms^5,29^. In contrast, all but one currently approved COVID-19 vaccines mediate protection through systemic virus neutralizing antibodies and likely also virus-specific T-cells, but induce only low mucosal IgA responses, as shown in a recent study of recipients of the BNT162b2 COVID-19 mRNA vaccine^30–32^. This lack of mucosal immunity was further confirmed by the observation that fully vaccinated index cases with breakthrough infections transmitted the virus to household contacts at a similar rate as unvaccinated index cases of approx. 25%^33^. The emergence of novel variants such as omicron indicates that SARS-COV-2 is continuing to mutate and evolve to subvert existing immune responses and increase transmissibility. Therefore, regular booster immunizations will be required to prevent severe disease and curb spread of the virus even in populations with a high level of pre-existing partial immunity, acquired either through vaccination or natural infection^34,35^. Second generation COVID-19 vaccines that confer broad protection, can easily be mass produced and deployed, and are user- friendly, offer the prospect of containing the virus in the long-term.

To this end, we designed a novel intranasal vaccine candidate based on the recombinantly expressed receptor binding domain (RBD) of SARS-CoV-2, fused to a polypeptide to specifically target antigen uptake by microfold cells (M-cells) of the nasal and bronchial-associated lymphoid tissue (NALT, BALT), and adjuvanted it with a novel toll-like receptor 3/RIG-I agonist consisting of short, double-stranded RNA.

The RBD, against which approx. 90% of the SARS-CoV-2 neutralizing antibody response is directed, has previously been identified as an ideal antigen for next generation Covid vaccines in multiple preclinical and clinical studies^36,37^. Since recombinant proteins typically induce no or only weak immune responses in the nasal mucosa^38^, we increased the immunogenicity by fusing the RBD to a short polypeptide derived from the C-terminus of the Clostridium perfringens *Clostridium perfringens *toxin (cCPE), which targets highly expressed claudin-4 on microfold cells (M-cells) and has previously been described to enhance the immunogenicity of intranasal pneumococcal and influenza vaccines^10,39^. M-cells are specialized epithelial cells, localized in mucosal crypts of the NALT and BALT, that carry sample antigen and transport it across the mucosa to adjacent antigen-presenting cells (APC), including dendritic cells, and are therefore attractive target cells for mucosal immunization^40–44^. Processed antigen is presented to CD4^+^ T cells in the lymphoid tissues that can induce IgA-committed B-cell development in the lymphoid follicle. Targeting the RBD to M-cells increased RBD-binding antibody titers of our vaccine in all compartments measured, with up to 11.9-fold and differences of GMTs between non-targeted and targeted RBD for IgG. Difference of pseudotype virus neutralizing antibody (IC_50_) reached statistical significance in BALF with highest titers in RBD-cCPE immunized animals, whereas in serum the IC_50_ trended higher in RBD-cCPE animals and showed much less variability, but the differences did not reach statistical significance due to small group sizes.

Spike-binding and pseudotyped-virus neutralizing systemic IgG antibody titers were equivalent to those induced in mice by two doses of mRNA or adenoviral vaccines, with similar neutralizing titers against wildtype Wuhan-Hu-1, the alpha variant B.1.1.7, and the delta variant B.1.617.2. We observed an approx. tenfold drop of neutralizing titers against the beta variant B.1.351, which has been also reported for mRNA vaccines^45^. The vaccine induced robust mucosal and systemic antibody responses and systemic CD4 and CD8 T-cell responses, when given either in as an intranasal prime/intranasal boost, or as a subcutaneous prime/intranasal boost.

In humans, most experience with experimental mucosal adjuvants has been gained with intranasal influenza vaccines adjuvanted with heat labile toxin of *E.coli* (LT), and toll-like receptor agonists such as monophosphoryl lipid A (TLR4), CpG (TLR9) and poly-IC:LC (TLR3)^46^. Only LT was used in a commercial product, however, that vaxccine (NasalFlu) to be withdrawn from the market due to an increased occurrence of Bell’s palsy^47^. The potential toxicity of mucosal adjuvants for intranasal application is therefore of concern and must be considered when developing intranasal vaccines. To this end, TLR3 adjuvants have been safely tested in non-human primates and in phase 1 trials in humans, both as influenza vaccine adjuvants as well as unspecific prophylactic monotherapy in human challenge models with influenza and rhinovirus^16,48^. Importantly, intranasal administration of TLR3 agonists as monotherapy in humans at more than 100-fold higher doses (up to 6.4 mg) than used in our study was not associated with safety or tolerability issues^15^. Interestingly, TLR3 is involved in mucosal IgA antibody induction not only in a CD4 T-cell dependent manner via stimulation of CD103^+^ dendritic cells, but also in a T-cell independent manner via direct stimulation of extrafollicular mucosal B-cells that were shown to undergo rapid class-switch recombination when stimulated with double-stranded viral RNA^49,50^. Riboxxim agonists may therefore be an ideal mucosal adjuvant for the induction of rapid IgA responses.

To further evaluate the versatility of our vaccine platform, we co-formulated a SARS-CoV-2 RBD / Influenza H1-HA_1_ vaccine, with both antigens targeted to M-cells, and adjuvanted with Riboxxim. A HA_1_ consensus sequence derived from 25 isolates of the influenza strain A/swine/Guangxi/3843/2011 (H1N1) was chosen, because G4 Eurasian (EA) avian-like H1N1 viruses have been spreading in pigs in China since 2016 and are considered a potential pandemic threat^51^. The combination vaccine induced high SARS-CoV-2 binding and neutralizing antibody titers in serum, NALF and BALF, including against the delta variant, which were very similar to those observed with the monovalent vaccine. In addition, the vaccine induced mucosal IgA and high systemic IgG antibodies binding monomeric recombinant HA_1_ protein. These data show that the combination of two different M-targeted vaccine antigens does not seem to result in immunodominance, which suggests that development of an intranasal vaccine protecting against multiple viruses may be feasible.

In summary, our novel vaccine platform overcomes the shortcomings of previous recombinant protein based intranasal vaccines by combining M-cell targeting of the vaccine antigen with a TLR3 agonist. This vaccine can be used for intranasal boosting following an intranasal or subcutaneous boost, induces systemic immune responses comparable to mRNA vaccines, is temperature stable, and can potentially be self-administered with an appropriate device. As such, it could play an important role in long-term containment of the virus.

## Supplementary Information

Supplementary Figure 1.

Supplementary Figure 2.

## Data Availability

The DNA sequences of the vaccine constructs have been deposited in GenBank under accession numbers: BankIt2646502 RBD OP896053; BankIt2646502 HA1-cCPE OP896054; BankIt2646502 RBD-cCPE OP896055.
