# Supplementary material for: Novel intranasal vaccine targeting SARS-CoV-2 receptor binding domain to mucosal microfold cells and adjuvanted with TLR3 agonist Riboxxim™ elicits strong antibody and T-cell responses in mice
Source: Sci Rep. 2023 Mar 21;13:4648. doi: 10.1038/s41598-023-31198-3 (PMC10029786; doi:10.1038/s41598-023-31198-3)
Supplement: Supplementary file 1 — Supplementary Figure 1. [file 41598_2023_31198_MOESM1_ESM.pptx]

## Slide 1
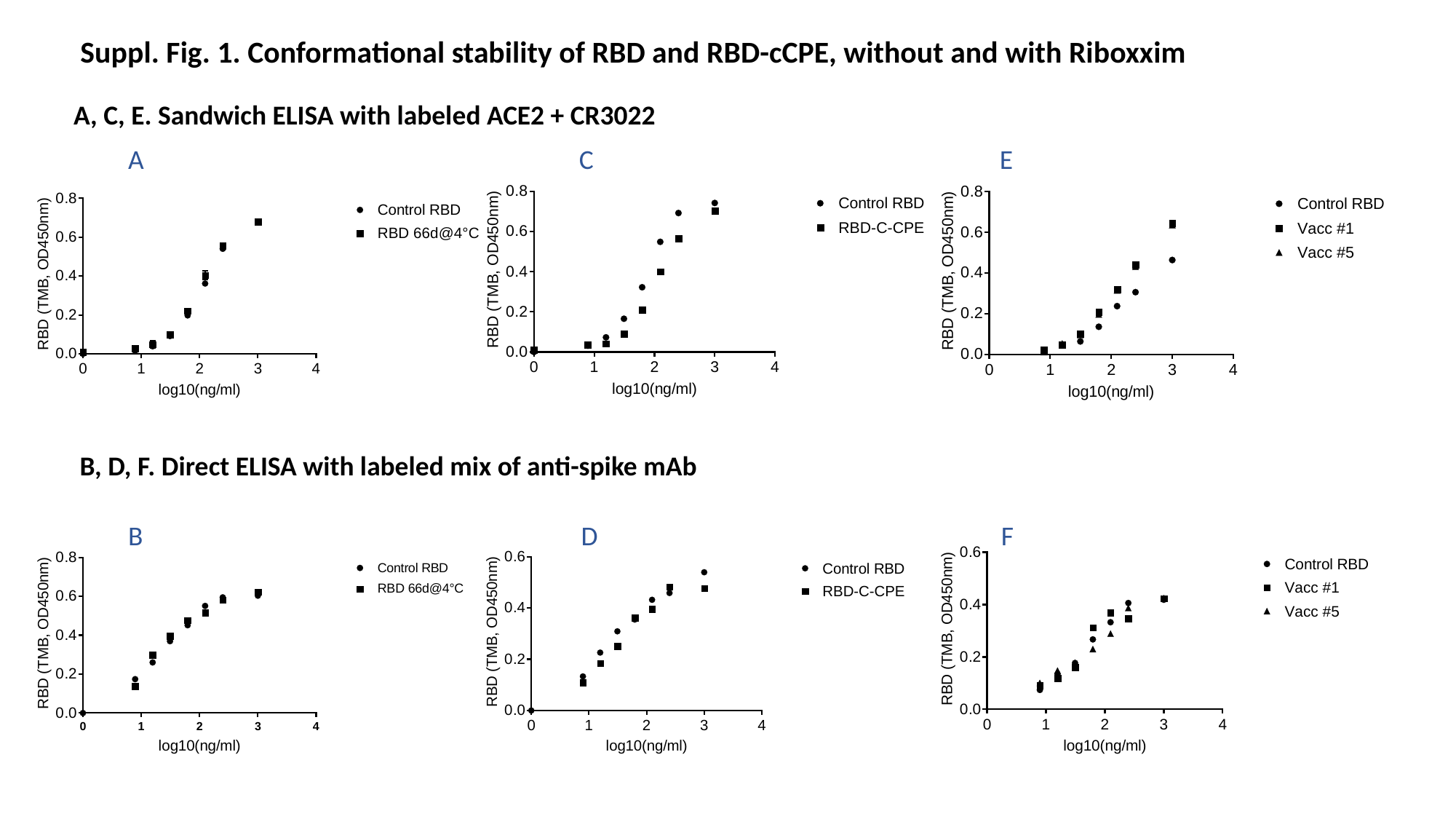

Suppl. Fig. 1. Conformational stability of RBD and RBD-cCPE, without and with Riboxxim
A, C, E. Sandwich ELISA with labeled ACE2 + CR3022
A
C
E
 B, D, F. Direct ELISA with labeled mix of anti-spike mAb
B
D
F

## Slide 2
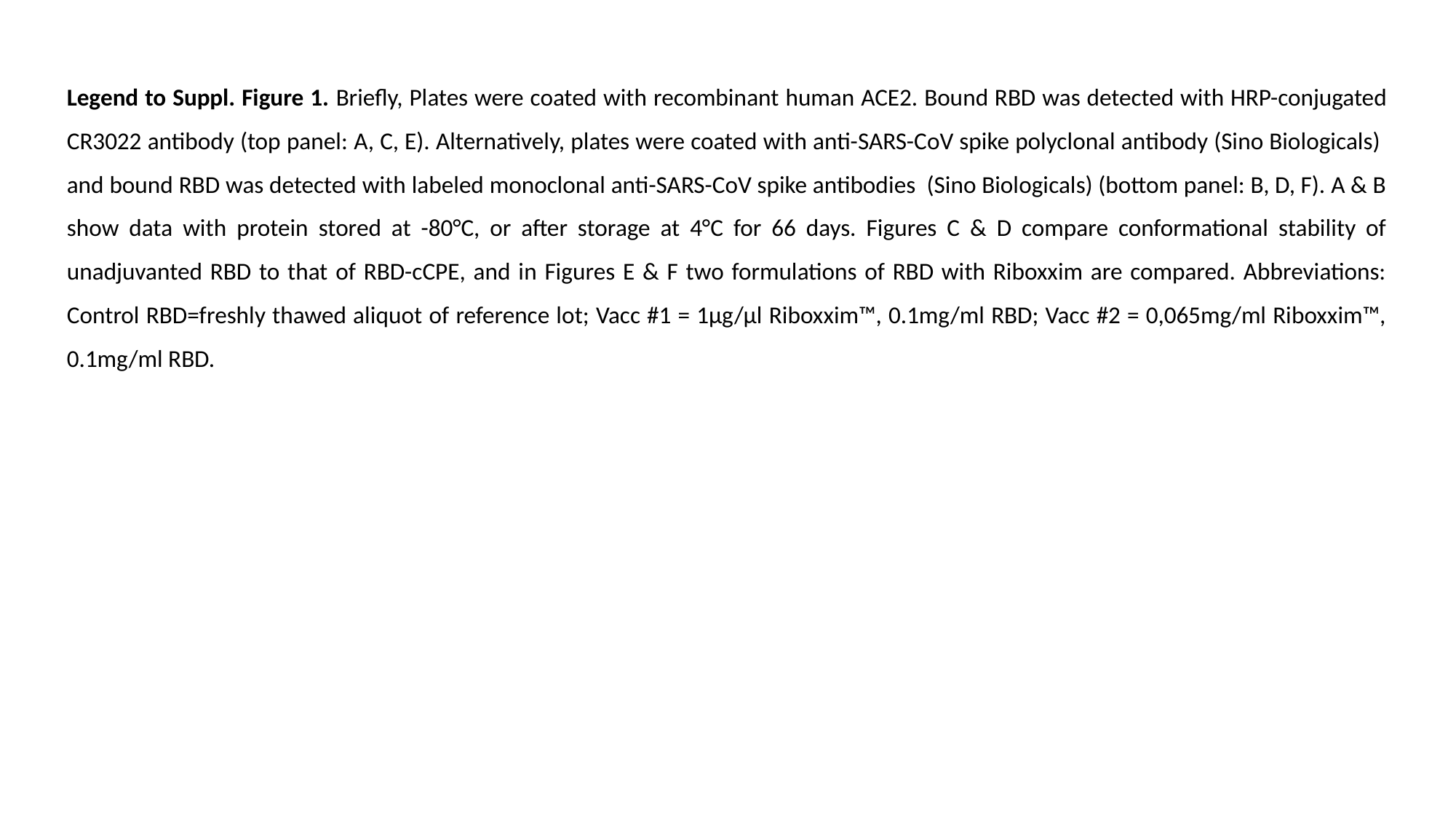

Legend to Suppl. Figure 1. Briefly, Plates were coated with recombinant human ACE2. Bound RBD was detected with HRP-conjugated CR3022 antibody (top panel: A, C, E). Alternatively, plates were coated with anti-SARS-CoV spike polyclonal antibody (Sino Biologicals) and bound RBD was detected with labeled monoclonal anti-SARS-CoV spike antibodies (Sino Biologicals) (bottom panel: B, D, F). A & B show data with protein stored at -80°C, or after storage at 4°C for 66 days. Figures C & D compare conformational stability of unadjuvanted RBD to that of RBD-cCPE, and in Figures E & F two formulations of RBD with Riboxxim are compared. Abbreviations: Control RBD=freshly thawed aliquot of reference lot; Vacc #1 = 1µg/µl Riboxxim™, 0.1mg/ml RBD; Vacc #2 = 0,065mg/ml Riboxxim™, 0.1mg/ml RBD.
