# Supplementary material for: Novel intranasal vaccine targeting SARS-CoV-2 receptor binding domain to mucosal microfold cells and adjuvanted with TLR3 agonist Riboxxim™ elicits strong antibody and T-cell responses in mice
Source: Sci Rep. 2023 Mar 21;13:4648. doi: 10.1038/s41598-023-31198-3 (PMC10029786; doi:10.1038/s41598-023-31198-3)
Supplement: Supplementary file 2 — Supplementary Figure 2. [file 41598_2023_31198_MOESM2_ESM.pptx]

## Slide 1
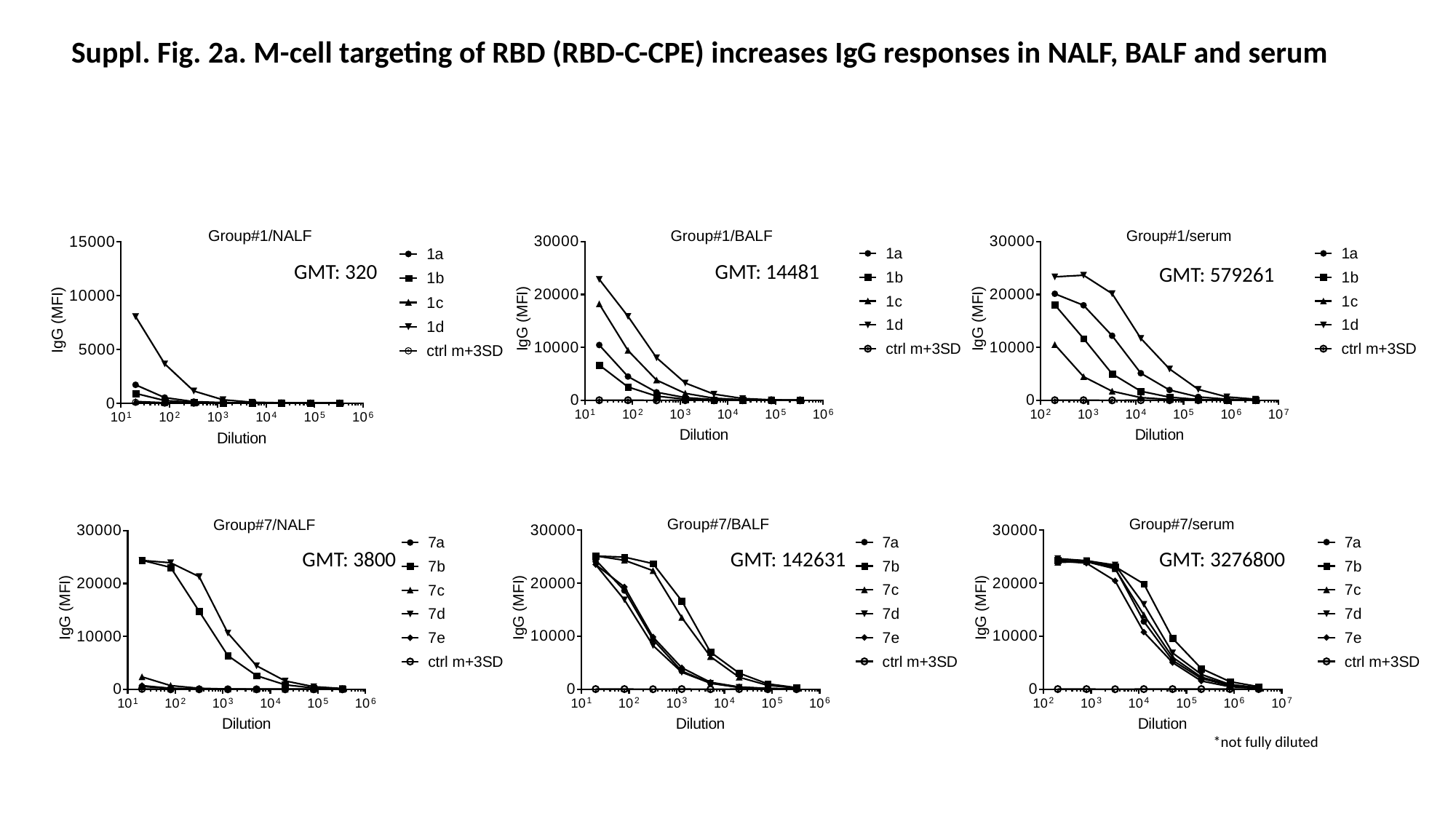

Suppl. Fig. 2a. M-cell targeting of RBD (RBD-C-CPE) increases IgG responses in NALF, BALF and serum
GMT: 320
GMT: 14481
GMT: 579261
GMT: 3800
GMT: 142631
GMT: 3276800
*not fully diluted

## Slide 2
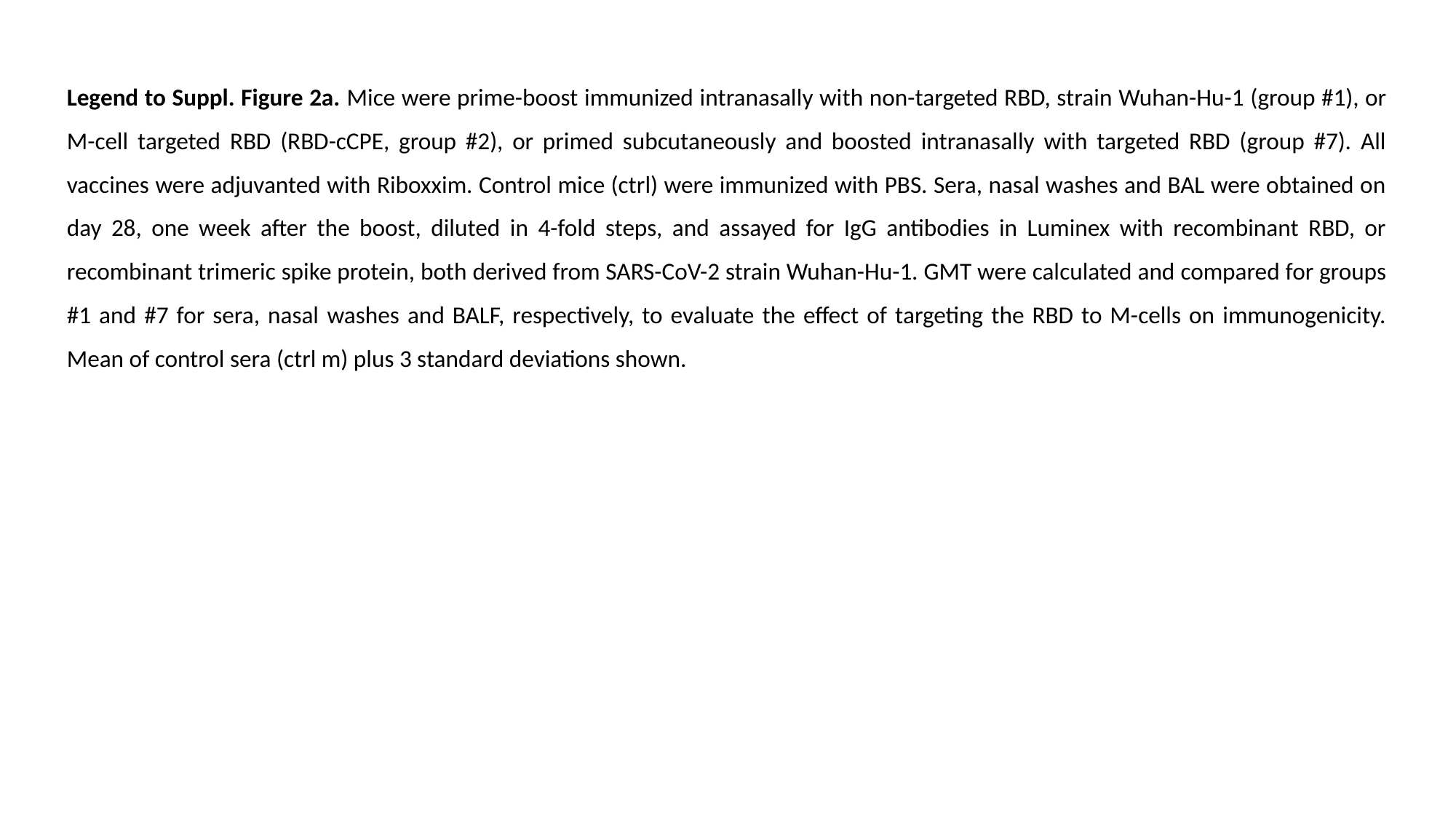

Legend to Suppl. Figure 2a. Mice were prime-boost immunized intranasally with non-targeted RBD, strain Wuhan-Hu-1 (group #1), or M-cell targeted RBD (RBD-cCPE, group #2), or primed subcutaneously and boosted intranasally with targeted RBD (group #7). All vaccines were adjuvanted with Riboxxim. Control mice (ctrl) were immunized with PBS. Sera, nasal washes and BAL were obtained on day 28, one week after the boost, diluted in 4-fold steps, and assayed for IgG antibodies in Luminex with recombinant RBD, or recombinant trimeric spike protein, both derived from SARS-CoV-2 strain Wuhan-Hu-1. GMT were calculated and compared for groups #1 and #7 for sera, nasal washes and BALF, respectively, to evaluate the effect of targeting the RBD to M-cells on immunogenicity. Mean of control sera (ctrl m) plus 3 standard deviations shown.

## Slide 3
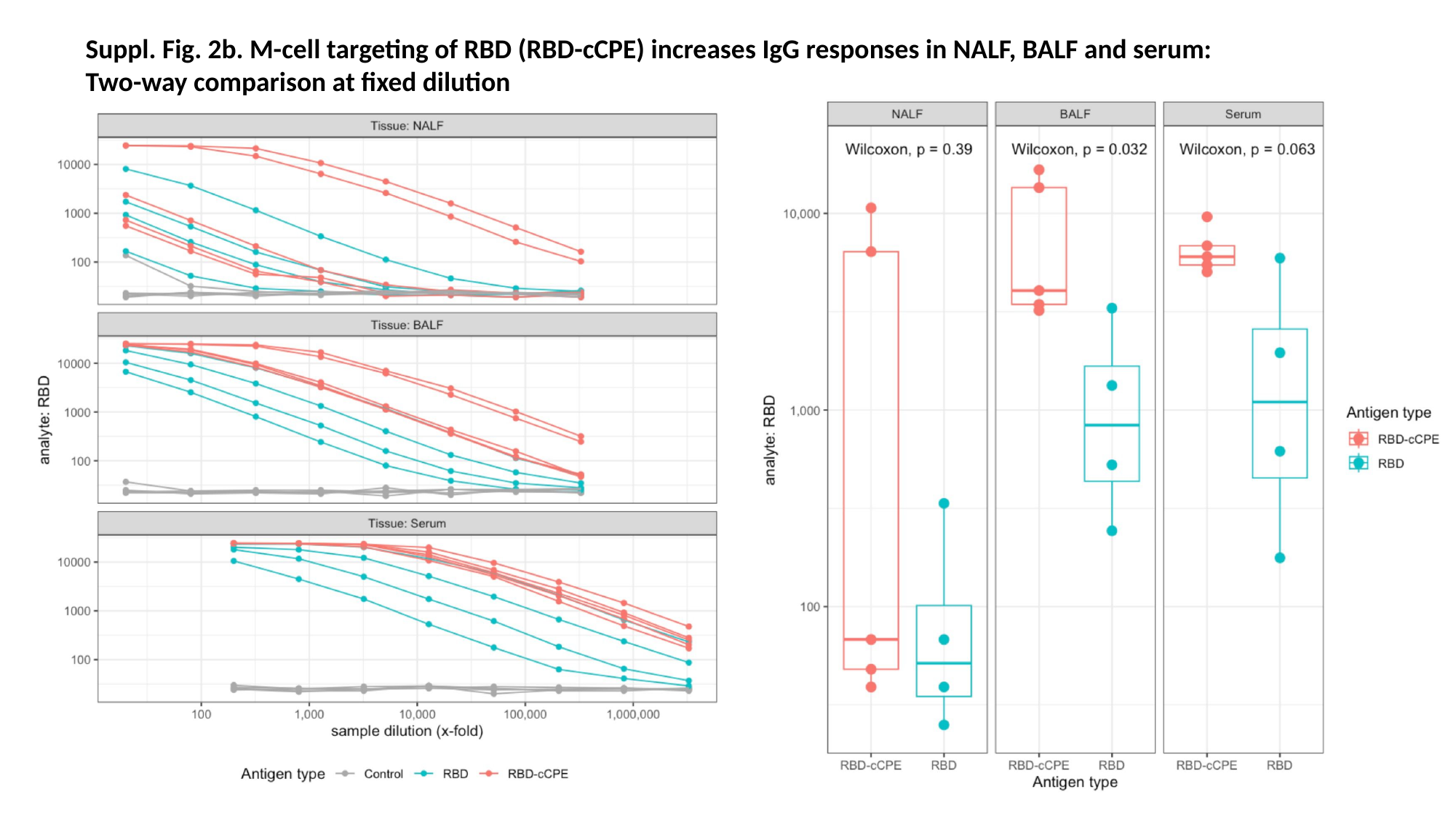

Suppl. Fig. 2b. M-cell targeting of RBD (RBD-cCPE) increases IgG responses in NALF, BALF and serum: Two-way comparison at fixed dilution

## Slide 4
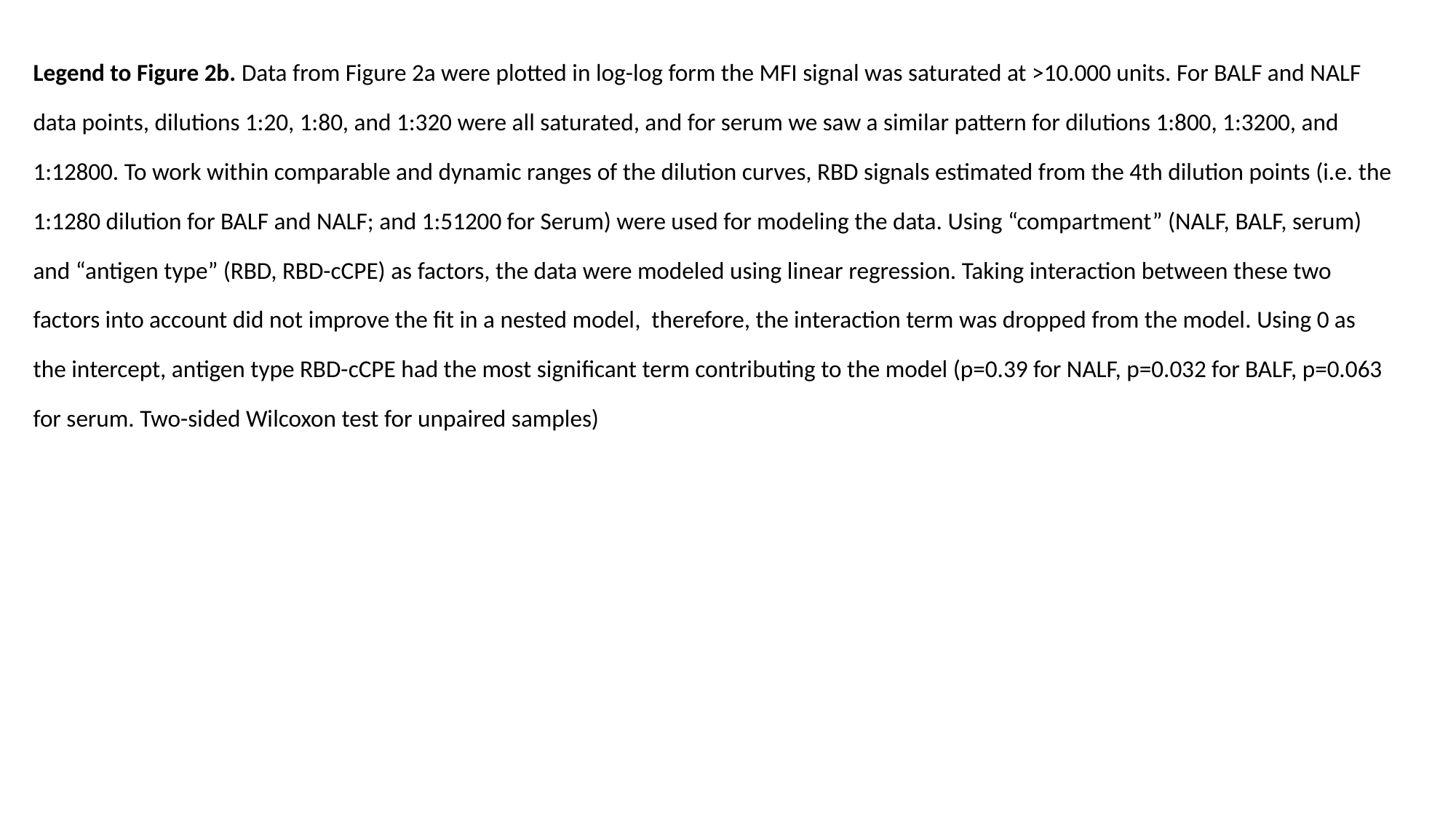

Legend to Figure 2b. Data from Figure 2a were plotted in log-log form the MFI signal was saturated at >10.000 units. For BALF and NALF data points, dilutions 1:20, 1:80, and 1:320 were all saturated, and for serum we saw a similar pattern for dilutions 1:800, 1:3200, and 1:12800. To work within comparable and dynamic ranges of the dilution curves, RBD signals estimated from the 4th dilution points (i.e. the 1:1280 dilution for BALF and NALF; and 1:51200 for Serum) were used for modeling the data. Using “compartment” (NALF, BALF, serum) and “antigen type” (RBD, RBD-cCPE) as factors, the data were modeled using linear regression. Taking interaction between these two factors into account did not improve the fit in a nested model, therefore, the interaction term was dropped from the model. Using 0 as the intercept, antigen type RBD-cCPE had the most significant term contributing to the model (p=0.39 for NALF, p=0.032 for BALF, p=0.063 for serum. Two-sided Wilcoxon test for unpaired samples)
